# Nasal drug delivery devices: characteristics and performance in a clinical perspective—a review

**DOI:** 10.1007/s13346-012-0108-9

**Published:** 2012-10-18

**Authors:** Per Gisle Djupesland

**Affiliations:** OptiNose, Oslo, Norway

**Keywords:** Drug delivery, Nasal, Device, Paranasal sinuses, Topical, Systemic, Vaccine, Nasal valve, Particle deposition, Clearance

## Abstract

Nasal delivery is the logical choice for topical treatment of local diseases in the nose and paranasal sinuses such as allergic and non-allergic rhinitis and sinusitis. The nose is also considered an attractive route for needle-free vaccination and for systemic drug delivery, especially when rapid absorption and effect are desired. In addition, nasal delivery may help address issues related to poor bioavailability, slow absorption, drug degradation, and adverse events in the gastrointestinal tract and avoids the first-pass metabolism in the liver. However, when considering nasal delivery devices and mechanisms, it is important to keep in mind that the prime purpose of the nasal airway is to protect the delicate lungs from hazardous exposures, not to serve as a delivery route for drugs and vaccines. The narrow nasal valve and the complex convoluted nasal geometry with its dynamic cyclic physiological changes provide efficient filtration and conditioning of the inspired air, enhance olfaction, and optimize gas exchange and fluid retention during exhalation. However, the potential hurdles these functional features impose on efficient nasal drug delivery are often ignored. With this background, the advantages and limitations of existing and emerging nasal delivery devices and dispersion technologies are reviewed with focus on their clinical performance. The role and limitations of the in vitro testing in the FDA guidance for nasal spray pumps and pressurized aerosols (pressurized metered-dose inhalers) with local action are discussed. Moreover, the predictive value and clinical utility of nasal cast studies and computer simulations of nasal airflow and deposition with computer fluid dynamics software are briefly discussed. New and emerging delivery technologies and devices with emphasis on Bi-Directional™ delivery, a novel concept for nasal delivery that can be adapted to a variety of dispersion technologies, are described in more depth.

## Introduction

Intuitively, the nose offers easy access to a large mucosal surface well suited for drug- and vaccine delivery. However, factors related to the nasal anatomy, physiology and aerodynamics that can severely limit this potential, have historically been challenging to address. The most recent FDA guidance for nasal devices provides detailed guidelines for in vitro testing of the physical properties such as in vitro reproducibility and accuracy of plume characteristics and dose uniformity of mechanical liquid spray pumps and pressurized metered-dose inhalers (pMDIs) for nasal use [1]. The guidance primarily addresses in vitro testing of nasal sprays and pressurized aerosols for local action. The reference to in vivo performance is limited to the recommendation of minimizing the fraction of respirable particles below 9 μm in order to avoid lung inhalation of drugs intended for nasal delivery. Thus, although important as measures of the quality and reliability of the spray pump and pMDI mechanics, these in vitro tests do not necessarily predict the in vivo particle deposition, absorption, and clinical response [2]. Furthermore, the guidance offers no or limited guidance on nasal products for systemic absorption and for alternative dispensing methods like drops, liquid jets, nebulized aerosol, vapors, and powder formulations. Finally, it does not address aspects and challenges related to the nasal anatomy and physiology that are highly relevant for the device performance in the clinical setting like body position, need for coordination, and impact of airflow and breathing patterns at delivery.

The mechanical properties of different modes of aerosol generation are already well described in depth in a previous publication [3]. The anatomy and physiology of the nasal airway has also recently been summarized in an excellent recent review [4]. The aim of this paper is to take a step further by reviewing the characteristics of existing and emerging nasal delivery devices and concepts of aerosol generation from the perspective of achieving the clinical promise of nasal drug and vaccine delivery. Focus is put on describing how the nasal anatomy and physiology present substantial obstacles to efficient delivery, but also on how it may be possible to overcome these hurdles by innovative approaches that permit realization of the therapeutic potential of nasal drug delivery. Specific attention is given to the particular challenge of targeted delivery of drugs to the upper narrow parts of the complex nasal passages housing the middle meatus where the sinuses openings are located, as well as the regions innervated by the olfactory nerve and branches of the trigeminal nerve considered essential for efficient “nose-to-brain” (N2B) transport.

## Nasal anatomy and physiology influencing drug delivery

### Regulation of nasal airflow

Nasal breathing is vital for most animals and also for human neonates in the first weeks of life. The nose is the normal and preferred airway during sleep, rest, and mild exercise up to an air volume of 20–30 l/min [5]. It is only when exercise becomes more intense and air exchange demands increase that oral breathing supplements nasal breathing. The switch from nasal to oronasal breathing in young adults appears when ventilation is increased to about 35 l/min, about four times resting ventilation [6]. More than 12,000 l of air pass through the nose every day [5]. The functionality of the nose is achieved by its complex structure and aerodynamics. Amazingly, the relatively short air-path in the nose accounts for as much as 50–75 % of the total airway resistance during inhalation [7, 8].

### The nasal valve and aerodynamics

The narrow anterior triangular dynamic segment of the nasal anatomy called the nasal valve is the primary flow-limiting segment, and extends anterior and posterior to the head of the inferior turbinate approximately 2–3 cm from the nostril opening [9]. This narrow triangular-shaped slit acts as a dynamic valve to modify the rate and direction of the airflow during respiration [10, 11]. Anatomical studies describe the static valve dimensions as 0.3–0.4 cm^2^ on each side, whereas acoustic rhinometry studies report the functional cross-sectional area perpendicular to the acoustic pathway to be between 0.5 and 0.6 cm^2^ on each side, in healthy adults, with no, or minimal gender differences [11–14]. The flow rate during tidal breathing creates air velocities at gale force (18 m/s) and can approach the speed of a hurricane (32 m/s) at sniffing [11, 15]. At nasal flow rates found during rest (up to 15 l/min), the flow regimen is predominantly laminar throughout the nasal passages. When the rate increases to 25 l/min, local turbulence occurs downstream of the nasal valve [10, 11, 15]. The dimensions can expand to increase airflow by dilator muscular action known as flaring, or artificially by mechanical expansion by internal or external dilators [16, 17]. During inhalation, Bernoulli forces narrow the valve progressively with increasing inspiratory flow rate and may even cause complete collapse with vigorous sniffing in some subjects [5]. During exhalation, the valve acts as a “brake” to maintain a positive expiratory airway pressure that helps keep the pharyngeal and lower airways open and increase the duration of the expiratory phase. This “braking” allows more time for gas exchange in the alveoli and for retention of fluid and heat from the warm saturated expiratory air [4, 17, 18]. In fact, external dilation of narrow noses in obstructive sleep apnea patients had beneficial effects, whereas dilation of normal noses to “supernormal” dimensions had deleterious effects on sleep parameters [17]. However, in the context of nasal drug delivery, the small dimensions of the nasal valve, and its triangular shape that narrows further during nasal inhalation, represent important obstacles for efficient nasal drug delivery.

### The nasal mucosa—filtration and clearance

**Fig. 1:**
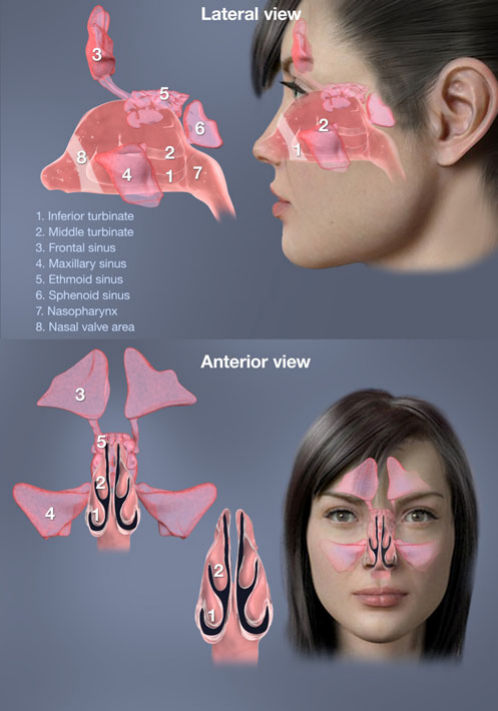
The complex anatomy of the nasal airways and paranasal sinuses

**Fig. 2:**
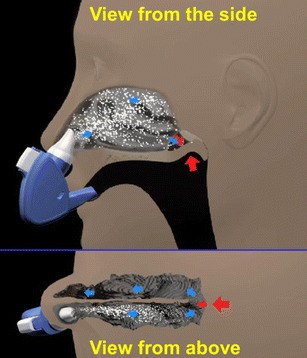
Illustration of the breath-powered Bi-Directional™ technology. See text for detailed description

**Fig. 3:**
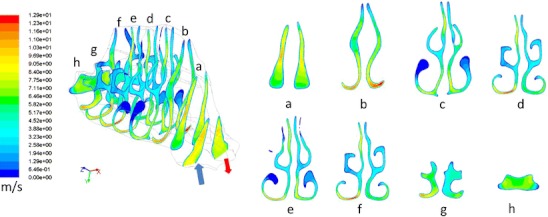
Cross-sections of a human nose with normal dimensions during soft palate closure with Bi-Directional™ flow assessment using CFD. The airflow is entering the right nostril and exiting the left nostril. The figure illustrates the narrow triangular shape of the nasal valve and the narrow slit-like passage of the nasal airway more posterior

The region anterior to the valve called the vestibule is lined by non-ciliated squamous epithelium that in the valve region gradually transitions into ciliated epithelium typical of the ciliated respiratory epithelium posterior to the valve region [4, 19]. Beyond the nasal valve, the nasal turbinates divide the nasal cavity into slit-like passages with much larger cross-sectional area and surface area (Figs. 1, 2 and 3). Here, the predominantly laminar airflow is slowed down to speeds of 2–3 m/s and disrupted with eddies promoting deposition of particles carried with the air at and just beyond the valve region [11]. The ciliated respiratory mucosa posterior to the nasal valve is covered by a protective mucous blanket designed to trap particles and microorganisms [4, 19].The beating action of cilia moves the mucous blanket towards the nasopharynx at an average speed of 6 mm/min (3–25 mm/min) [20, 21]. The large surface area and close contact enables effective filtering and conditioning of the inspired air and retention of water during exhalation (Figs. 1, 2 and 3). Oral breathing increases the net loss of water by as much as 42 % compared to nasal breathing [22]. The nasal passages were optimized during evolution to protect the lower airways from the constant exposure to airborne pathogens and particles. Specifically, particles larger than 3–10 μm are efficiently filtered out and trapped by the mucus blanket [19]. The nose also acts as an efficient “gas mask” removing more that 99 % of water-soluble, tissue-damaging gas like sulfur dioxide [23]. Infective agents are presented to the abundant nasal immune system both in the mucous blanket, in the mucosa, and in the adjacent organized lymphatic structures making the nose attractive for vaccine delivery with potential for a longstanding combination of systemic and mucosal immune responses [24]. The highly vascularized respiratory mucosa found beyond the valve allows exchange of heat and moisture with the inspired air within fractions of a second, to transform cold winter air into conditions more reminiscent of a tropical summer [19].

### The nasal cycle

The physiological alternating congestion and decongestion observed in at least 80 % of healthy humans is called the nasal cycle [5, 25]. The nasal cycle was first described in the rhinological literature by a German physician in 1895, but was recognized in Yoga literature centuries before [5]. Healthy individuals are normally unaware of the spontaneous and irregular reciprocal 1–4-h cycling of the nasal caliber of the two individual passages, as the total nasal resistance remains fairly constant [26]. The autonomic cyclic change in airflow resistance is mainly dependent on the blood content of the submucosal capacitance vessels that constitute the erectile component at critical sites, notably the nasal valve region. Furthermore, the erectile tissues of the septal and lateral walls and the turbinates respond to a variety of stimuli including physical and sexual activity and emotional states that can modify and override the basic cyclic rhythm [4]. The cycle is present during sleep, but overridden by pressures applied to the lateral body surface during recumbency to decongest the uppermost/contralateral nasal passage. It has been suggested that this phenomenon causes a person to turn from one side to the other while sleeping [5, 27]. The cycle is suppressed in intubated subjects, but restored by resumption of normal nasal breathing [28]. The cycle may also cause accumulation of nitric oxide (NO) in the congested passage and adjacent sinuses and contribute to defense against microbes through direct antimicrobial action and enhanced mucociliary clearance [29]. Measurements have shown that the concentration of NO in the inspired air is relatively constant due to the increase in NO concentration within the more congested cavity, which nearly exactly counterbalances the decrease in nasal airflow [30]. In some patients, as a result of structural deviations and inflammatory mucosal swelling, the nasal cycle may become clinically evident and cause symptomatic obstruction [19]. Due to the cycle, one of the nostrils is considerably more congested than the other most of the time, and the vast majority of the airflow passes through one nostril while the other remains quite narrow especially at the valve region [5]. Consequently, the nasal cycle contributes significantly to the dynamics and resistance in the nasal valve region and must be taken into consideration when the efficiency of nasal drug delivery devices is considered.

### Nasal and sinus vasculature and lymphatic system

For nasally delivered substances, the site of deposition may influence the extent and route of absorption along with the target organ distribution. Branches of the ophthalmic and maxillary arteries supply the mucous membranes covering the sinuses, turbinates, meatuses, and septum, whereas the superior labial branch of the facial artery supplies the part of the septum in the region of the vestibule. The turbinates located at the lateral nasal wall are highly vascularized with a very high blood flow and act as a radiator to the airway. They contain erectile tissues and arteriovenous anastomoses that allow shunting and pooling related to temperature and water control and are largely responsible for the mucosal congestion and decongestion in health and disease [19, 31].

Substances absorbed from the anterior regions are more likely to drain via the jugular veins, whereas drugs absorbed from the mucosa beyond the nasal valve are more likely to drain via veins that travel to the sinus cavernous, where the venous blood comes in direct contact with the walls of the carotid artery. A substance absorbed from the nasal cavity to these veins/venous sinuses will be outside the blood–brain barrier (BBB), but for substances such as midazolam, which easily bypass the BBB, this route of local “counter-current transfer” from venous blood may provide a faster and more direct route to the brain. Studies in rats support that a preferential, first-pass distribution to the brain through this mechanism after nasal administration may exist for some, but not all small molecules [32, 33]. The authors suggested that this counter-current transport takes place in the area of the cavernous sinus–carotid artery complex, which has a similar structure in rat and man, but the significance of this mechanism for nasally delivered drugs has not been demonstrated in man [32, 33].

The lymphatic drainage follows a similar pattern as the venous drainage where lymphatic vessels from the vestibule drain to the external nose to submandibular lymph nodes, whereas the more posterior parts of the nose and paranasal sinuses drain towards the nasopharynx and internal deep lymph nodes [4]. In the context of nasal drug delivery, perivascular spaces along the olfactory and trigeminal nerves acting as lymphatic pathways between the CNS and the nose have been implicated in the transport of molecules from the nasal cavity to the CNS [34].

### Innervation of the nasal mucosa

The nose is also a delicate and advanced sensory organ designed to provide us with the greatest pleasures, but also to warn and protect us against dangers. An intact sense of smell plays an important role in both social and sexual interactions and is essential for quality of life. The sense of smell also greatly contributes to taste sensations [35]. Taste qualities are greatly refined by odor sensations, and without the rich spectrum of scents, dining and wining and life in general would become dull [36]. The olfactory nerves enter the nose through the cribriform plate and extend downwards on the lateral and medial side of the olfactory cleft. Recent biopsy studies in healthy adults suggest that the olfactory nerves extend at least 1–2 cm further anterior and downwards than the 8–10 mm described in most textbooks (see Figs. 1 and 2) [37, 38]. The density decreases, but olfactory filaments and islets with olfactory epithelium are found in both the anterior and posterior parts at the middle turbinate. In addition, sensory fibers of both the ophthalmic and maxillary branches of the trigeminal nerve contribute to olfaction by mediating a “common chemical sense” [39]. Branches of the ophthalmic branch of the trigeminal nerve provide sensory innervation to the anterior part of the nose including the vestibule, whereas maxillary branches innervate the posterior part of the nose as well as the regions with olfactory epithelium.

The olfactory and trigeminal nerves mutually interact in a complex manner. The trigeminal system can modulate the olfactory receptor activity through local peptide release or via reflex mechanisms designed to minimize the exposure to and effects of potentially noxious substances [39]. This can occur by alteration of the nasal patency and airflow and through changes in the properties of the mucous blanket covering the epithelium. Trigeminal input may amplify odorous sensation through perception of nasal airflow and at the chemosensory level. Interestingly, an area of increased trigeminal chemosensitivity is found in the anterior part of the nose, mediating touch, pressure, temperature, and pain [39]. Pain receptors in the nose are not covered by squamous epithelium, which gives chemical stimuli almost direct access to the free nerve endings. In fact, loss of trigeminal sensitivity and function, and not just olfactory nerve function, may severely reduce the sense of smell [40]. This should not be forgotten when addressing potential causes of reduced or altered olfaction.

### The sensitivity of the nasal mucosa as a limiting factor

In addition to the limited access, obstacles imposed by its small dimensions and dynamics, the high sensitivity of the mucosa in the vestibule and in the valve area is very relevant to nasal drug delivery. Direct contact of the tip of the spray nozzle during actuation, in combination with localized concentrated anterior drug deposition on the septum, may create mechanical irritation and injury to the mucosa resulting in nosebleeds and crusting, and potentially erosions or perforation [41]. Furthermore, the high-speed impaction and low temperature of some pressurized devices may cause unpleasant sensations reducing patient acceptance and compliance.

The role of the high sensitivity of the nasal mucosa as a natural nasal defense is too often neglected when the potential of nasal drug delivery is discussed, in particular when results from animal studies, cast studies, and computer fluid dynamics (CFD) are evaluated. Exposure to chemicals, gases, particles, temperature and pressure changes, as well as direct tactile stimuli, may cause irritation, secretion, tearing, itching, sneezing, and severe pain [39]. Sensory, motor, and parasympathetic nerves are involved in a number of nasal reflexes with relevance to nasal drug delivery [4]. Such sensory inputs and related reflexes are suppressed by the anesthesia and/or sedation often applied to laboratory animals, potentially limiting the clinical predictive value of such studies. Further, the lack of sensory feedback and absence of interaction between the device and human subjects/patients are important limitations of in vitro testing of airflow and deposition patterns in nasal casts and in CFD simulation of deposition. Consequently, deposition studies in nasal casts and CFD simulation of airflow and deposition are of value, but their predictive value for the clinical setting are all too often overestimated.

### Targeted nasal delivery

For most purposes, a broad distribution of the drug on the mucosal surfaces appears desirable for drugs intended for local action or systemic absorption and for vaccines [3]. However, in chronic sinusitis and nasal polyposis, targeted delivery to the middle and superior meatuses where the sinus openings are, and where the polyps originate, appears desirable [42, 43]. Another exception may be drugs intended for “nose-to-brain” delivery, where more targeted delivery to the upper parts of the nose housing the olfactory nerves has been believed to be essential. However, recent animal data suggest that some degree of transport can also occur along the branches of the first and second divisions of the trigeminal nerve innervating most of the mucosa at and beyond the nasal valve [44]. This suggests that, in contrast to the prevailing opinion, a combination of targeted delivery to the olfactory region and a broad distribution to the mucosa innervated by the trigeminal nerve may be optimal for N2B delivery. Targeted delivery will be discussed in more detail below.

## Nasal drug delivery devices

The details and principles of the mechanics of particle generation for the different types of nasal aerosols have been described in detail by Vidgren and Kublik [3] in their comprehensive review from 1998 and will only be briefly described here, with focus instead on technological features directly impacting particle deposition and on new and emerging technologies and devices. Liquid formulations currently completely dominate the nasal drug market, but nasal powder formulations and devices do exist, and more are in development. Table 1 provides an overview of the main types of liquid and powder delivery devices, their key characteristics, and examples of some key marketed nasal products and emerging devices and drug–device combination products in clinical development (Table 1).

**Table 1:**
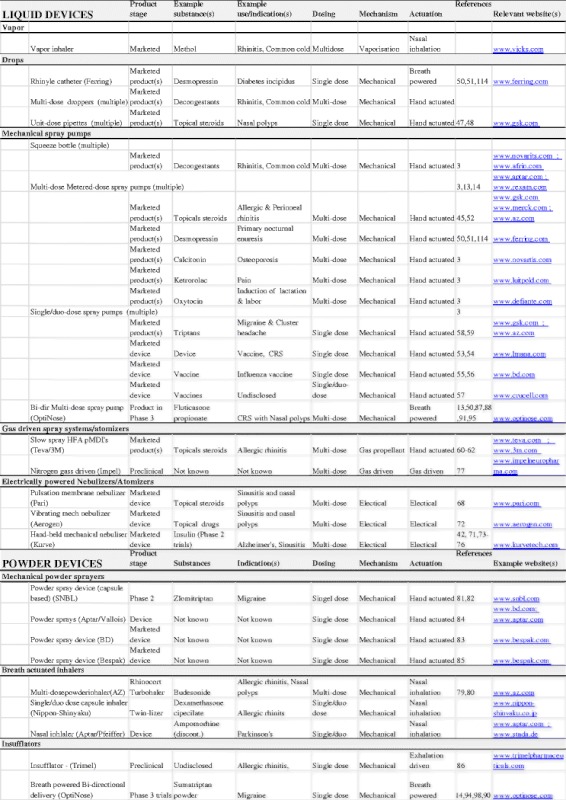
Overview of the main types of liquid and powder delivery devices, their key characteristics, and examples of some key marketed nasal products and emerging devices and drug–device combination products in clinical development

### Devices for liquid formulations

The liquid nasal formulations are mainly aqueous solutions, but suspensions and emulsions can also be delivered. Liquid formulations are considered convenient particularly for topical indications where humidification counteracts the dryness and crusting often accompanying chronic nasal diseases [3]. In traditional spray pump systems, preservatives are typically required to maintain microbiological stability in liquid formulations. Studies in tissue cultures and animals have suggested that preservatives, like benzalkonium chloride in particular, could cause irritation and reduced ciliary movement. However, more recent human studies based on long-term and extensive clinical use have concluded that the use of benzalkonium chloride is safe and well tolerated for chronic use [45]. For some liquid formulations, in particular peptides and proteins, limited stability of dissolved drug may represent a challenge [46].

#### Drops delivered with pipette

Drops and vapor delivery are probably the oldest forms of nasal delivery. Dripping breast milk has been used to treat nasal congestion in infants, vapors of menthol or similar substances were used to wake people that have fainted, and both drops and vapors still exist on the market (e.g., www.vicks.com). Drops were originally administered by sucking liquid into a glass dropper, inserting the dropper into the nostril with an extended neck before squeezing the rubber top to emit the drops. For multi-use purposes, drops have to a large extent been replaced by metered-dose spray pumps, but inexpensive single-dose pipettes produced by “blow-fill-seal” technique are still common for OTC products like decongestants and saline. An advantage is that preservatives are not required. In addition, due to inadequate clinical efficacy of spray pumps in patients with nasal polyps, a nasal drop formulation of fluticasone in single-dose pipettes was introduced in the EU for the treatment of nasal polyps. The rationale for this form of delivery is to improve drug deposition to the middle meatus where the polyps emerge [47, 48]. However, although drops work well for some, their popularity is limited by the need for head-down body positions and/or extreme neck extension required for the desired gravity-driven deposition of drops [43, 49]. Compliance is often poor as patients with rhinosinusitis often experience increased headache and discomfort in head-down positions.

#### Delivery of liquid with rhinyle catheter and squirt tube

A simple way for a physician or trained assistant to deposit drug in the nose is to insert the tip of a fine catheter or micropipette to the desired area under visual control and squirt the liquid into the desired location. This is often used in animal studies where the animals are anesthetized or sedated, but can also be done in humans even without local anesthetics if care is taken to minimize contact with the sensitive mucosal membranes [50]. This method is, however, not suitable for self-administration. Harris et al. [51] described a variant of catheter delivery where 0.2 ml of a liquid desmopressin formulation is filled into a thin plastic tube with a dropper. One end of the tube is positioned in the nostril, and the drug is administered into the nose as drops or as a “liquid jet” by blowing through the other end of the thin tube by the mouth [51]. Despite a rather cumbersome procedure with considerable risk of variability in the dosing, desmopressin is still marketed in some countries with this rhinyle catheter alongside a nasal spray and a tablet for treatment of primary nocturnal enuresis, Von Willebrand disease, and diabetes insipidus.

#### Squeeze bottles

Squeeze bottles are mainly used to deliver some over-the-counter (OTC) products like topical decongestants. By squeezing a partly air-filled plastic bottle, the drug is atomized when delivered from a jet outlet. The dose and particle size vary with the force applied, and when the pressure is released, nasal secretion and microorganisms may be sucked into the bottle. Squeeze bottles are not recommended for children [3].

#### Metered-dose spray pumps

Metered spray pumps have, since they were introduced some four decades ago, dominated the nasal drug delivery market (Table 1). The pumps typically deliver 100 μl (25–200 μl) per spray, and they offer high reproducibility of the emitted dose and plume geometry in in vitro tests. The particle size and plume geometry can vary within certain limits and depend on the properties of the pump, the formulation, the orifice of the actuator, and the force applied [3]. Traditional spray pumps replace the emitted liquid with air, and preservatives are therefore required to prevent contamination. However, driven by the studies suggesting possible negative effects of preservatives, pump manufacturers have developed different spray systems that avoid the need for preservatives. These systems use a collapsible bag, a movable piston, or a compressed gas to compensate for the emitted liquid volume [3] (www.aptar.com and www.rexam.com). The solutions with a collapsible bag and a movable piston compensating for the emitted liquid volume offer the additional advantage that they can be emitted upside down, without the risk of sucking air into the dip tube and compromising the subsequent spray. This may be useful for some products where the patients are bedridden and where a head-down application is recommended. Another method used for avoiding preservatives is that the air that replaces the emitted liquid is filtered through an aseptic air filter. In addition, some systems have a ball valve at the tip to prevent contamination of the liquid inside the applicator tip (www.aptar.com). These preservative-free pump systems become more complex and expensive, and since human studies suggest that preservatives are safe and well tolerated, the need for preservative-free systems seems lower than previously anticipated [45]. More recently, pumps have been designed with side-actuation and introduced for delivery of fluticasone furoate for the indication of seasonal and perennial allergic rhinitis [52]. The pump was designed with a shorter tip to avoid contact with the sensitive mucosal surfaces. New designs to reduce the need for priming and re-priming, and pumps incorporating pressure point features to improve the dose reproducibility and dose counters and lock-out mechanisms for enhanced dose control and safety are available (www.rexam.com and www.aptar.com). Importantly, the in vivo deposition and clinical performance of metered-dose spray pumps can be enhanced for some applications by adapting the pumps to a novel breath-powered “Bi-Directional™” delivery technology described in more detail below [13].

#### Single- and duo-dose spray devices

Metered-dose spray pumps require priming and some degree of overfill to maintain dose conformity for the labeled number of doses. They are well suited for drugs to be administered daily over a prolonged duration, but due to the priming procedure and limited control of dosing, they are less suited for drugs with a narrow therapeutic window. For expensive drugs and vaccines intended for single administration or sporadic use and where tight control of the dose and formulation is of particular importance, single-dose or duo-dose spray devices are preferred (www.aptar.com).

A simple variant of a single-dose spray device (MAD) is offered by LMA (LMA, Salt Lake City, UT, USA; www.lmana.com). A nosepiece with a spray tip is fitted to a standard syringe. The liquid drug to be delivered is first drawn into the syringe and then the spray tip is fitted onto the syringe. This device has been used in academic studies to deliver, for example, a topical steroid in patients with chronic rhinosinusitis and in a vaccine study [53, 54]. A pre-filled device based on the same principle for one or two doses (Accuspray™, Becton Dickinson Technologies, Research Triangle Park, NC, USA; www.bdpharma.com) is used to deliver the influenza vaccine FluMist (www.flumist.com), approved for both adults and children in the US market [55, 56]. A similar device for two doses was marketed by a Swiss company for delivery of another influenza vaccine a decade ago. This vaccine was withdrawn due to occurrence of adverse events (Bell’s palsy) potentially related to the cholera toxin adjuvant used [57]. The device technology is now owned by a Dutch vaccine company (Crucell N.V. Leiden, the Netherlands; www.crucell.com), but to our knowledge is not currently used in any marketed products.

The single- and duo-dose devices mentioned above consist of a vial, a piston, and a swirl chamber. The spray is formed when the liquid is forced out through the swirl chamber. These devices are held between the second and the third fingers with the thumb on the actuator. A pressure point mechanism incorporated in some devices secures reproducibility of the actuation force and emitted plume characteristics [58]. Currently, marketed nasal migraine drugs like Imitrex (www.gsk.com) and Zomig (www.az.com; Pfeiffer/Aptar single-dose device) and the marketed influenza vaccine FluMist (www.flumist.com; Becton Dickinson single-dose spray device) are delivered with this type of device [59] (Table 1). With sterile filling, the use of preservatives is not required, but overfill is required resulting in a waste fraction similar to the metered-dose, multi-dose sprays. To emit 100 μl, a volume of 125 μl is filled in the device (Pfeiffer/Aptar single-dose device) used for the intranasal migraine medications Imitrex (sumatriptan) and Zomig (zolmitriptan) and about half of that for a duo-dose design [58].

#### Nasal pressurized metered-dose inhalers (pMDIs)

Most drugs intended for local nasal action are delivered by spray pumps, but some have also been delivered as nasal aerosols produced by pMDIs. Following the ban on ozone-depleting chlorofluorocarbon (CFC) propellants, the number of pMDI products for both pulmonary and nasal delivery diminished rapidly, and they were removed from the US market in 2003 [60]. The use of the old CFC pMDIs for nasal products was limited due to complaints of nasal irritation and dryness. The particles from a pMDI are released at a high speed and the expansion of a compressed gas, which causes an uncomfortable “cold Freon effect” [61]. The particles emitted from the traditional pMDIs had a particle velocity much higher than a spray pump (5,200 vs. 1,500 cm/s at a distance 1–2 cm from the actuator tip) [3]. The issues related to the high particle speed and “cold Freon effect” have been reduced with the recently introduced hydrofluoroalkane (HFA)-based pMDI for nasal use offering lower particle speeds [60]. Recently, the first nasal pMDI using HFA as propellant to deliver the first generation topical steroid beclomethasone dipropionate (BDP) was approved for allergic rhinitis in the USA [62]. Like spray pumps, nasal pMDIs produce a localized deposition on the anterior non-ciliated epithelium of the nasal vestibule and in the anterior parts of the narrow nasal valve, but due to quick evaporation of the spray delivered with a pMDI, noticeable “drip-out” may be less of an issue [63].

#### Mismatch between geometry of anterior nose and the spray plume

The pressure created by the force actuating a spray pump drives the liquid through the swirl chamber at the tip of the applicator and out through the circular nozzle orifice [64]. The combination of radial and axial forces creates a swirling thin sheet of liquid that, after some millimeters, becomes unstable and breaks up into “ligaments” before forming the particles (break-up length). Importantly, a hollow spray cone is formed with particles mainly at the periphery. The key parameters influencing the properties of the plume and subsequently the deposition pattern of the particles are the swirl effect, nozzle orifice dimensions, the spray cone angle, and the break-up length. Inthavong et al. [64] reported for a spray with a nozzle diameter of 0.5 mm, a spray cone angle of 30°, and a break-up length of about 3.5 mm, and the diameter at the break-up point is already 4 mm. One study reported the smallest spray cone diameters (*D*_max_/*D*_min_) for a spray angle with 54.6° to be 2.34/1.92 and 3.30/3.08 cm at distances of 1.0 and 2.5 cm from the nozzle [2]. Another study reported a spray cone diameter of 2.52/1.58 at 3 cm from the nozzle for a spray angle of 39° [65]. Even if the spray pump is inserted as deep as 10–15 mm into the nostril, there is an obvious mismatch between the dimensions and shape of the circular plume (diameter≈2 cm) and the narrow triangular valve opening. With most of the particles in the periphery of the plume, it becomes quite evident that the majority of the particles will impinge in the non-ciliated mucosal walls of the vestibule anterior to the valve. Particles actually penetrating the valve will do so primarily through the lower and wider part of the triangle, a delivery pattern that is accentuated if delivery is performed during sniffing. Although the aerosol-generating mechanisms are different, a similar mismatch would exist between constricting geometry of the nasal vestibule and the conical-shaped plumes produced by other powered devices like pMDIs, nebulizers/atomizers, and many powder devices (see below).

##### Powered nebulizers and atomizers

Nebulizers use compressed gasses (air, oxygen, and nitrogen) or ultrasonic or mechanical power to break up medical solutions and suspensions into small aerosol droplets that can be directly inhaled into the mouth or nose. The smaller particles and slow speed of the nebulized aerosol are advocated to increase penetration to the target sites in the middle and superior meatuses and the paranasal sinuses [42]. Indeed, nasal inhalation from a nebulizer has been shown to improve deposition to the upper narrow part of the nose when compared to a metered-dose spray pump, but with 33 % and 56 % of the delivered dose deposited in the lungs in the subjects assessed [66]. In light of this problem of lung delivery, it is unsurprising that nasal inhalation of nebulized antibiotics intended for topical action in patients with chronic rhinosinusitis resulted in coughing and increased need for inhaled medications following nasal inhalation [67].

##### VibrENT pulsation membrane nebulizer

A new nebulizer intended for delivery to the nose and sinuses in patients with chronic rhinosinusitis utilizing a pulsating aerosol generated via a perforated vibrating membrane has recently been introduced (VibrENT PARI Pharma GmbH). The pulsation in combination with small particles is assumed to offer better penetration to the sinuses, and instruction on specific breathing technique during delivery is advocated to minimize inhalation [68]. Delivery of an aerosol with small particles with a mass median aerodynamic diameter (MMAD) of 3.0 μm was performed with two different techniques and compared to a spray pump. Aerosol administration into one nostril for 20 s at a rate of mass output of 0.3 ml/min, with an exit filter attached to the other nostril during nasal breathing, resulted in 4.5 % of the fraction deposited in the nose (63 %) reaching the sinuses (i.e., 2.8 % of the delivered dose), 27 % in the exit filter, and significant lung deposition (10 %). Nasal aerosol delivery was also performed when the subjects were instructed to maintain the soft palate closed while a flow resistor was connected to the left nostril. Following this procedure, 70 % of the radioactivity was deposited in the nose, 30 % in the exit filter, a negligible fraction in the lungs, and 7 % of the fraction in the nose (i.e., 4.9 % of the delivered dose) was found in the sinuses [68]. Following delivery of 100 μl with a traditional spray pump, 100 % of the dose was found in the nose with no deposition in the lungs and non-significant deposition in the sinuses [68]. Correction for background radiation and decay was performed, but correction for tissue attenuation was not performed, which is likely to change the relative distribution and potentially increase the fraction actually deposited in the lungs [68–71]. Nevertheless, the results suggest that the use of a pulsating aerosol in combination with the breathing technique and an exit resistor may enhance deposition in the sinuses in healthy volunteers. However, the clinical relevance of these results from healthy volunteers for rhinosinusitis patients with blocked sinus openings remains to be determined. The proposed breathing technique used to prevent lung deposition may also prove challenging as compared to the automatic integration of velum closure and the drug delivery process, as achieved when using the exhalation breath in operation of the delivery device, such as provided by OptiNose’s Bi-Directional™ delivery technology, which can also utilize an exit resistor to create positive pressure in the nose and sinuses[69]. Furthermore, a very distinct “hot spot” was observed for both the nebulizer and spray pump delivery, but no assessment of regional deposition in the nose was performed in the study with the pulsating aerosol nebulizer [68].

##### Aeroneb Solo vibrating mesh nebulizer

Distinct anterior deposition in the valve area with nebulizers is confirmed in another very recent publication comparing nasal inhalation from a nasal sonic/pulsating jet nebulizer (Atomisor NL11S^®^ sonic, DTF-Medical, France) and a new nasal mesh nebulizer system designed to minimize lung inhalation (Aeroneb Solo^®^, Aerogen, Galway, Ireland; DTF-Aerodrug, Tours, France) with the same mean particle size (5.6 ± 0.5 μm) [72]. The new system consists of two integrated components: the nebulizer compressor administering a constant airflow rate transporting the aerosol into one nostril via a nozzle and a pump simultaneously aspirating from a second nozzle in the other nostril at the same airflow rate while the subject is instructed to avoid nasal breathing [72]. The new nasal mesh nebulizer produced more deposition in terms of volume of liquid (27 % vs. 9 %, i.e., 0.81 vs. 0.27 ml) in the nasal cavity. The much higher fraction found in the nasal cavity in this study is probably a result of the shorter nebulizing time and smaller delivered volume in the study testing the PARI pulsating nebulizer (20 s at a rate of 0.3 ml/min to each nostril versus delivery of 3 ml for up to 10 min) before assessment of deposition was performed [68, 72]. With much longer delivery time, a substantial fraction of the dose delivered beyond the nasal valve will be cleared to the gastrointestinal (GI) tract.

Aerosol distribution deposition showed a distinct maximum value at 2 cm from the nostril for both nebulizers corresponding to deposition in the nasal valve region [72]. Furthermore, aerosol distribution deposition in the vertical plane showed a similar profile for both nebulizers with a distinct maximum close to the floor of the nose (0.75 cm for the mesh nebulizer and 1.2 cm for the sonic jet nebulizer) [72]. Importantly, the delivery efficiencies for both nebulizers and delivery techniques appear very low with only 27 % vs. 9 %, i.e., 0.81 vs. 0.27 ml, possibly due to the long delivery time and resulting differences in mucociliary and other mechanisms of clearance [72]. In other words, a study assessing deposition after several minutes of delivery is likely to underestimate the actual exposure to the posterior ciliated part of the nose compared to the study assessing deposition after a short period of delivery of less than 1 min (20 s × 2) [68, 72].

##### Clinical relevance of deposition results with nebulizers

Lung deposition and relatively low nasal delivery fractions are issues with nasal nebulizers. Although lung deposition appears to be reduced with simultaneous aspiration from the contralateral nostril and with specific breathing instructions, this complex mechanism for use, coupled with the need for careful patient compliance with breathing, may be challenging, especially in children or other special populations [66, 68, 72]. The study design, comparing not only two different nebulization techniques but also very different breathing techniques, makes interpretation of the results comparing the nasal nebulizers in terms of deposition efficacy and clinical significance very difficult.

The rationale for using small particles and sonic/pulsation techniques is to increase the delivery into the sinuses, but at the expense of low delivery efficacy and significant potential for lung deposition. Moreover, despite the intended advantages of the vibrating mesh nebulizer that employs aspiration from the contralateral nostril, the quantification of deposition in the different planes (cartography) demonstrates the typical highly preferential deposition in the anterior (anterior 2–3 cm) and lower (lower 1–2 cm) parts of the nasal cavity. This pattern of deposition suggests the nebulizer is not effectively delivering to the prime target sites for chronic rhinosinusitis and nasal polyposis (i.e., the middle and superior meatuses or sinuses) [42, 72]. To date, no clinical data has been published with the new nebulizer systems [68, 72].

One approach to avoiding lung deposition is the Bi-Directional^TM^ technology employed in OptiNose devices; this technology ensuring operation of the nebulizer only on generation of a pressure sufficient to close the palate, avoiding the problems associated with suction pumps and special breathing instructions. However, clinical data using this approach with a nebulizer has also not been published.

##### ViaNase atomizer

A handheld battery-driven atomizer intended for nasal drug delivery has been introduced (ViaNase by Kurve Technology Inc., Lynnwood, WA, USA). This device atomizes liquids by producing a vortical flow on the droplets as they exit the device (www.kurvetech.com). The induced vortical flow characteristics can be altered in circular velocity and direction to achieve different droplet trajectories [42, 73]. As discussed above, it is not clear that vortex flow is desirable for penetration past the nasal valve; however, it has been suggested that this technology is capable of targeting the sinuses, and some gamma-deposition images suggesting delivery to the sinuses have been published. However, no information related to impact of prior surgery or numerical quantification of nasal or sinus deposition verifying the claimed improved deposition to the upper parts of the nose has been published [42, 73]. The ViaNase device has been used to deliver nasal insulin in patients with early Alzheimer’s disease (AD), and clinical benefit has been demonstrated [74, 75]. In these studies, delivery of insulin was performed over a 2-min period by nasal inhalation. However, when insulin is delivered with this device, lung deposition is likely to occur, and some concerns related to airway irritation and reduction in pulmonary function have been raised in relation to long-term exposure to inhaled insulin when Exubera was marketed for a short period as a treatment for diabetes [71, 76]. This example highlights the issue of unintended lung delivery, one important potential clinical problem associated with using nebulizers and atomizers producing respirable particles for nasal drug delivery.

##### Impel nitrogen-driven atomizer

A nasal atomizer driven by highly pressurized nitrogen gas is under development by Impel Inc. (www.impel.com). The device is intended to enable drug delivery to the upper parts of the nose in order to achieve N2B delivery [77]. To date, only animal data has been presented, making it difficult to evaluate its potential in human use, as nasal deposition and the assessment of nasal deposition in animal models vary significantly from humans. As previously noted, however, pMDIs are associated with a number of limitations. It therefore remains to be seen if a pressurized “open-palate” nebulizer will be capable of creating the desired delivery pattern.

### Powder devices

Powder medication formulations can offer advantages, including greater stability than liquid formulations and potential that preservatives may not be required. Powders tend to stick to the moist surface of the nasal mucosa before being dissolved and cleared. The use of bioadhesive excipients or agents that slow ciliary action may decrease clearance rates and improve absorption [46, 78]. A number of factors like moisture sensitivity, solubility, particle size, particle shape, and flow characteristics will impact deposition and absorption [3].

The function of nasal powder devices is usually based on one of three principles (Table 1):

1. Powder sprayers with a compressible compartment to provide a pressure that when released creates a plume of powder particles fairly similar to that of a liquid spray;
2. Breath-actuated inhalers where the subject uses his own breath to inhale the powder into the nostril from a blister or capsule; and
3. Nasal insufflators describe devices consisting of a mouthpiece and a nosepiece that are fluidly connected. Delivery occurs when the subject exhales into the mouthpiece to close the velum, and the airflow carries the powder particles into the nose through the device nosepiece similar to the rhinyle catheter described above. The principle can be applied to different dispersion technologies and has been further developed and extended into the breath-powered Bi-Directional™ delivery technology (see below).

#### Nasal powder inhalers

- Astra Zenaca markets budesonide powder delivered with the Turbuhaler multi-dose inhaler device modified for nasal inhalation (Rhinocort Turbuhaler^®^; www.az.com) [79]. It is marketed for allergic rhinitis and nasal polyps in some markets as an alternative to the liquid spray, but it does not seem to offer any particular advantage [80]. In a study comparing twice daily treatment with aqueous budesonide spray (128 μg × 2) and the Rhinocort Turbuhaler^®^ (140 μg × 2) in nasal polyp patients, both treatments significantly reduced polyp size compared to placebo, but with no difference between the active treatments. However, nasal symptom scores were significantly more reduced in the liquid spray compared to the powder [80]. A gamma-deposition study with Rhinocort Turbuhaler) has shown predominantly anterior deposition with a “hot spot” at the nasal valve area and about 5 % lung deposition [79]. If corrected for tissue attenuation in the lungs, it is likely that the fraction would be substantially higher [69, 79].
- Aptar group (www.aptar.com) offers a simple blister-based powder inhaler. The blister is pierced before use and the device nosepiece placed into one nostril. The subject closes the other nostril with the finger and inhales the powder into the nose. A powder formulation of apomorphine for Parkinson’s using this blister-based powder inhaler (BiDose™/Prohaler™) from Pfeiffer/Aptar was in clinical development by Britannia, a UK company recently acquired by Stada Pharmaceutical (www.stada.de). Apparently, further development has been discontinued.
- Nippon Shinyaku Co., Ltd. (www.nippon-shinyaku.co.jp) markets in Japan a topical steroid (dexamethasone cipecilate) delivered with a powder-based inhalation device for allergic rhinitis. The device (Twin-lizer™) has two chambers with capsules inside. The capsule is pierced, and when the subject inhales from the nosepiece, the powder is deagglomerated and delivered into the nose with the airflow.

#### Nasal powder sprayers

- SBNL Pharma (www.snbl.com) recently reported data on a Phase 1 study described in a press release (www.snbl.com) with a zolmitriptan powder cyclodextrin formulation (μco™ System) for enhanced absorption, described previously in an in vitro study [81]. The zolmitriptan absorption was rapid, and the relative bioavailability was higher than the marketed tablet and nasal spray (www.snbl.com). The company has their own capsule-based, single-dose powder devices (Fit-lizer) [82]. When inserted into a chamber, the top and bottom of the capsule is cut off by sharp blades. A plastic chamber is compressed by hand, compressed air passes through a one-way valve and the capsule during actuation, and the powder is emitted. In vitro testing shows high-dose reproducibly and minimal residuals, but no data on particle size distribution or in vivo deposition and clearance patterns appear to be available. The company has also completed a Phase 2 study with the drug granisetron for the indication of delayed chemotherapy-induced nausea and vomiting based on the same formulation technology and delivered with the Fit-lizer™ device [81]. They have also announced plans to develop a powder-based influenza vaccine (www.snbl.com).
- Bespak (www.bespak.com), the principle for Unidose-DP™, is similar to the Fit-lizer device. An air-filled compartment is compressed until a pin ruptures a membrane to release the pressure to emit the plume of powder. Delivery of powder formulations of a model antibody (human IgG) has been tested in a nasal cast model based on human MRI images. Approximately 95 % of the dose was delivered to the nasal cavity, but the majority of it was deposited no further than the nasal vestibule with only about 30 % deposited into deeper compartments of the nasal cavity [83]. The company report in their website that they have entered into a collaboration to develop an undisclosed nasal powder product with this device (www.bespak.com).
- Aptar group (Pfeiffer/Valois) (www.aptar.com) offers a powder device (Monopowder) based on the same principle as the devices above but with a plunger that when pressed creates a positive pressure that ruptures a membrane to expel the powder. The device has been used in studies in rabbits, but no data from human deposition or clinical studies have been published [84].
- BD (www.bdpharma.com) also has a powder device (SoluVent™) where a positive pressure is created with a plunger that pierces a membrane to expel the powder. A device based on this technology is being tested with powder vaccines [85].

#### Nasal powder insufflators

- Trimel (www.trimel.com) has acquired a device originally developed by a Danish company (Direct Haler). There are two versions of this device that looks like a small drinking straw. One version is intended for pulmonary drug delivery where subjects inhale through the small tubular device and one for nasal drug delivery where subjects blow into one end of the tube while the other end is inserted into the vestibule of the nostril. The device can in principle be viewed as a powder version of the rhinyle catheter for liquid delivery. This tubular device includes a middle section with corrugations. The corrugations allow flexion of the device and create turbulence that deagglomerates the powder. One end of the small tubular device is inserted between the lips and the other into the nasal vestibule. The subject then exhales through the device to expel the powder from the tube and into the nostril. As when using the rhinyle catheter, exhalation into the device causes the soft palate to automatically elevate to separate the oral cavity and the nasal passages, preventing lung inhalation during delivery. No clinical data with the device is available apart from a small gamma study in a patent stating that the device produced clearance and areas of deposition that were not significantly different from a “state-of-the-art” powder inhalation device (device details not identified) [86].
- OptiNose (www.optinose.com) has developed a breath-powered Bi-Directional™ nasal delivery technology for liquid and powder medications which utilizes the exhaled breath to deliver the drug into the nose, but with additional key distinguishing features that importantly impact drug deposition and clearance patterns and clinical device performance.

#### Breath-powered Bi-Directional™ technology—a new nasal drug delivery concept

This novel concept exploits natural functional aspects of the upper airways to offer a delivery method that may overcome many of the inherent limitations of traditional nasal devices. Importantly, the breath-powered Bi-Directional™ technology can be adapted to any type of dispersion technology for both liquids and powders. Breath-powered Bi-Directional™ devices consist of a mouthpiece and a sealing nosepiece with an optimized frusto-conical shape and comfortable surface that mechanically expands the first part of the nasal valve (Figs. 1, 2, and 3). The user slides a sealing nosepiece into one nostril until it forms a seal with the flexible soft tissue of the nostril opening, at which point, it mechanically expands the narrow slit-shaped part of the nasal triangular valve. The user then exhales through an attached mouthpiece. When exhaling into the mouthpiece against the resistance of the device, the soft palate (or velum) is automatically elevated by the positive oropharyngeal pressure, isolating the nasal cavity from the rest of the respiratory system. Owing to the sealing nosepiece, the dynamic pressure that is transferred from the mouth through the device to the nose further expands the slit-like nasal passages. Importantly, the positive pressure in the entry nostril will, due to the sealing nosepiece, balance the oropharyngeal pressure across the closed velum to prevent the velum from being “over-elevated,” thus securing an open flow path between the two nasal passages behind the nasal septum and in front of the elevated velum.

This “breath-powered” mechanism enables release of liquid or powder particles into an air stream that enters one nostril, passes entirely around the nasal septum, and exits through the opposite nostril, following a “Bi-Directional™” flow path. Actuation of drug release in devices employing this approach has been described using manual triggering as well as mechanisms automatically triggered by flow and/or pressure [13, 69, 70, 87, 88]. By optimizing design parameters, such as the nosepiece shape, the flow rate, the particle size profile, and release angle, it is possible to optimize delivery to target sites beyond the nasal valve, avoid lung deposition, and to assure that particles are deeply deposited without exiting the contralateral nostril. The Bi-Directional™ devices currently in phase 3 clinical trials are a multi-dose liquid device incorporating a standard spray pump and a capsule-based powder multi-use device with disposable drug chamber and nosepiece (Fig. 3), but other configurations are possible. Importantly, the Bi-Directional™ delivery concept can be adapted to a variety of dispersion technologies for both liquids and powders,

##### Human evidence for nasal deposition patterns with Bi-Directional™ delivery

**Fig. 4:**
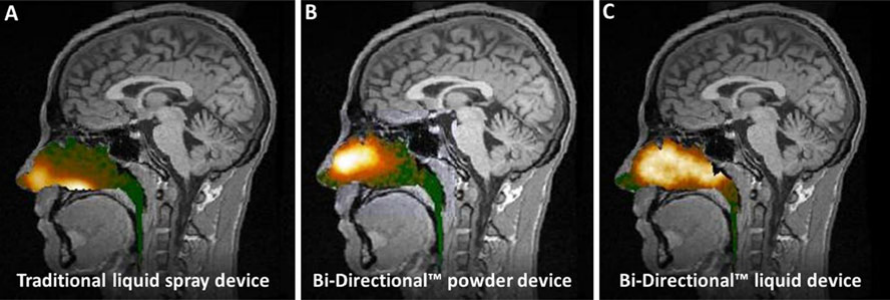
Gamma camera image information (logarithmic “hot iron” intensity scale) from the nasal cavity is superimposed on the corresponding sagittal MRI section. The images are from the same subject and present deposition 2 min after delivery using (**a**) a traditional liquid spray, (**b**) the breath-powered Bi-Directional™ powder device, and (**c**) the breath-powered Bi-Directional™ liquid spray device incorporating the same spray pump as used in **a**. The initial deposition following traditional spray was greatest in the lower anterior regions of the nose, whereas deposition with the Bi-Directional™ delivery devices was greatest in the upper posterior regions of the nose. The less broad distribution in **b** following breath-powered Bi-Directional™ powder device is believed to be due to the slower clearance for powder the first 6–8 min, reflecting the dissolution of the powder into the mucosal layer. **a** and **b** have been published previously, and they are reprinted with permission from the publisher [14]

Device variants using this mechanism of nasal drug delivery have been tested in gamma-deposition studies where assessments of the regional deposition and clearance patterns in human subjects were studied in detail [13, 14, 69]. Comparison of conventional nasal inhalation and Bi-Directional™ delivery with the same nebulizer producing small particles showed that lung inhalation can be prevented with Bi-Directional™ delivery even when small respirable particle are delivered [69]. In one published study, a breath-actuated Bi-Directional™ device incorporating a standard spray pump was compared directly to the same nasal spray pump actuated by hand in the traditional way, and in a second published study, a Bi-Directional™ powder device was directly compared to a traditional spray device [13, 14]. Both studies demonstrated less deposition in the non-ciliated nasal vestibule and significantly greater deposition to the upper posterior regions beyond the nasal valve with the Bi-Directional™ devices as compared to conventional delivery with a spray pump [13, 14] (Fig. 4). In the most recent gamma study with Bi-Directional™ powder device (Opt-Powder) seen in Fig. 2, the initial deposition in the upper and middle posterior regions of the nose was significantly larger than a traditional spray (upper posterior region; Opt-Powder 18.3 ± −11.5 % vs. spray 2.4 ± 1.8 %, *p* < 0.02; sum of upper and middle posterior regions; Opt-Powder 53.5 ± 18.5 % vs. spray 15.7 ± 13.8 %, *p* < 0.02) [14]. In contrast, the summed initial deposition to the lower anterior and posterior regions for spray was three times higher compared to Opt-Powder (Opt-Powder 17.4 ± 24.5 % vs. spray 59.4 ± 18.2 %, *p* < 0.04; Fig. 4) [14].

##### Published clinical outcomes with breath-powered Bi-Directional™ delivery devices

In addition to human studies of deposition patterns, devices using the breath-powered Bi-Directional™ technology have also been evaluated in a number of clinical trials. Results generally suggest that superior deep nasal deposition with clinically important potential can be achieved in the clinic, and two drug–device combinations are currently in Phase 3 development: sumatriptan powder for acute migraine and fluticasone propionate for chronic rhinosinusitis with nasal polyposis [87–90] (www.optinose.com).

- Midazolam—sedation: Midazolam is a drug with high bioavailability (BA), reasonable ability to cross the BBB, and easily observed pharmacodynamic effects (sedation). In a three-way crossover study of 12 healthy volunteers, delivery of the same dose of midazolam (3.4 mg) with a breath-powered Bi-Directional™ device prototype was assessed relative to a standard nasal spray and intravenous (IV) administration [91]. Drug pharmacokinetics (PK) with both nasal delivery approaches were similar, as is not unexpected for a small molecule easily absorbed to the blood with a high BA of ≈70 %. Interestingly, the pharmacodynamic effects (onset and level of sedation) reported with Bi-Directional™ delivery were very similar to IV administration despite substantially lower maximum serum levels (Bi-Directional™ with median *C*_max_ = 3 Ng/ml vs. IV with median *C*_max_ = 5 ng/ml). In contrast, the onset was slower, and the degree of sedation was lower following traditional spray delivery despite similar PK values as Bi-Directional™ delivery [91]. These findings suggest that the sedative effect following Bi-Directional™ nasal delivery may not merely be a result of absorption to the blood and subsequent passage into the brain across the BBB as occurs with a standard nasal spray. Alternative transport routes to the brain bypassing the BBB described in animal studies may contribute to the sedative effects [32–34, 44]. Absorption from the posterior part of the nose may offer a more direct route to brain arterial blood through the particular venous drainage pathway from the posterior parts of the nose called “counter-current transfer” [32, 33]. Moreover, direct transport to the brain for both small and large molecules may occur along ensheathed cells forming channels around the olfactory and trigeminal nerves [34, 44]. Contribution from such alternative transport routes would be consistent with a clinically important improvement in the pattern of deep nasal drug deposition with breath-powered Bi-Directional™ delivery (Fig. 4) [13, 14].
- Sumatriptan—migraine: Unlike midazolam, the serotonin antagonist sumatriptan has poor BA when delivered orally (14 %) and is only marginally higher when delivered as a nasal spray (Pfeiffer single-dose device). It has been estimated that only about 10 % of the drug delivered by standard nasal spray (Imitrex) is absorbed rapidly across the nasal mucosa within the first 20 min with much of a dose undergoing delayed absorption from the GI tract with a *T*_max_ of 90 min [92, 93]. Hypothesizing that breath-actuated Bi-Directional™ powder delivery may produce clinically different results than previously reported for nasal spray delivery, investigators conducted a cross-over PK study in 12 migraineurs, comparing subcutaneous injection of 6 mg sumatriptan with 10 and 20 mg of intranasal sumatriptan powder. Bi-directionally delivered nasal sumatriptan powder was pharmacodynamically similar to injection, inducing a similar EEG profile and preventing migraine attacks in patients when delivered 15 min before glyceryl trinitrate challenge. The PK curves showed a similar bi-phasic absorption pattern as described for sumatriptan nasal spray delivery, but with a substantially higher initial predominantly nasal absorption peak at 20 min estimated to account for approximately 30 % of the total absorption which is about three times the estimated 10 % fraction absorbed nasally for the marketed Imitrex nasal spray [89, 92]. These PK results lend credence to the conclusion that clinically differentiated nasal deposition is produced by the breath-powered Bi-Directional™ device compared to what has been previously reported with standard nasal spray delivery. A more definitive study directly comparing sumatriptan delivery with a breath-powered Bi-Directional™ device to delivery by standard nasal spray, oral delivery, and injection delivery is being conducted and should report results soon (www.clinicaltrials.gov). In a randomized, double-blind, parallel group, placebo-controlled study, a single migraine attack was treated in-clinic with two doses of sumatriptan powder (7.5 or 15 mg delivered doses or placebo) administered intranasally by a novel Bi-Directional™ powder delivery device; fast onset of pain relief was observed for both doses [90]. The pain relief rates were similar to historical data SC injection despite much lower systemic exposure [90, 92]. The results suggest that the enhanced deposition associated with the breath-powered Bi-Directional™ delivery of sumatriptan powder may contribute to greater initial nasal absorption and offer clinical benefits [94]. However, based on comparisons with historical data on the PK and pharmacodynamics profiles of sumatriptan delivered through different routes, it has been speculated that the rate of systemic absorption of nasal sumatriptan may not alone explain differences in headache response suggesting the potential for an additional route to the site of action as discussed above [14] . A Phase 3 study is currently in progress (www.clinicaltrials.gov and www.optinose.com).
- Fluticasone propionate—chronic rhinosinusitis with nasal polyps: Fluticasone is a topical steroid, available as a standard nasal spray for treatment of rhinitis but often used with limited benefit in the treatment of chronic rhinosinusitis (CRS) with and without nasal polyps. In a 3-month placebo controlled study in 109 patients with chronic rhinosinusitis (CRS) with nasal polyps, delivery of fluticasone (400 μg b.i.d.) with an OptiNose breath-powered Bi-Directional™ liquid drug delivery device was reported to be well tolerated and to produce a large magnitude of reduction in both symptoms and the overall polyp score. Particularly notable relative to expectations with standard nasal spray delivery, complete elimination of the polyps in close to 20 % of the subjects was reported after 3 months [87]. The proportion of subjects with improvement in summed polyp score was significantly higher with OptiNose fluticasone propionate (Opt-FP) compared with placebo at 4, 8, and 12 weeks (22 % vs. 7 %, *p* = 0.011, 43 % vs. 7 %, *p* < 0.001, 57 % vs. 9 %, *p* < 0.001). Despite relatively lower baseline polyp scores after 12 weeks, the summed polyp score was significantly reduced from 2.8 to 1.8 in the active treatment group, whereas a minor increase in polyp score was seen in the placebo group (−0.98 vs. +0.23, *p* < 0.001). Peak nasal inspiratory flow (PNIF) increased progressively during Opt-FP treatment (*p* < 0.001). Combined symptom score, nasal blockage, discomfort, rhinitis symptoms, and sense of smell were all significantly improved [87]. The highly significant progressive treatment effect of Opt-FP was observed regardless of baseline polyps score. Previous sinus surgery had no impact on the efficacy. Coupled with the complete removal of polyps in many patients with small polyps, this suggests that improved deposition to target sites achieved with the Bi-Directional™ delivery device may translate into true clinical benefits and possibly reduced need for surgery [95]. A Phase 3 study is currently in progress (www.clinicaltrials.gov and www.optinose.com).
  The same drug–device combination product was also evaluated in a small placebo controlled study (*N* = 20) in patients with post-surgical recalcitrant CRS without polyps, producing clinically significant improvements on both objective measures and subjective symptoms [88]. Endoscopy score for edema showed a significant and progressive improvement [12 weeks (median scores): Opt-FP −4.0, PBO −1.0, *p* = 0.015]. PNIF increased significantly during Opt-FP treatment compared to placebo (4 weeks: *p* = 0.006; 8 weeks: *p* = 0.03). After 12 weeks, MRI scores in the Opt-FP group improved against baseline (*p* = 0.039), and a non-significant trend was seen vs. placebo. The nasal RSOM-31 subscale was significantly improved with Opt-FP treatment (4 weeks: *p* = 0.009, 8 weeks: *p* = 0.016, 12 weeks: NS). Sense of smell, nasal discomfort, and combined score were all significantly improved (*p* < 0.05). Notably, this is a condition marked by many recent negative placebo-controlled trials [96, 97]. This context, in addition to comparison with historical data in similar patient populations, again suggests that breath-powered bi-directional delivery is capable of producing superior deep nasal deposition in clinical practice (improved targeting of the middle meatus in this case) which can translate into improved clinical response (Fig. 4) [13, 87, 88].
- Influenza vaccine: In a four-armed parallel group study with a whole-virus influenza liquid vaccine without adjuvant, delivery with the breath-powered Bi-Directional™ OptiNose device and nasal drops were found to provide better overall immune response than a traditional nasal spray and an oral spray [50]. In contrast to the self-administration with the OptiNose device, the nasal drops were delivered by an assistant inserting the pipette tip in a controlled manner beyond the nasal valve with the neck extended. These results suggest that Bi-Directional™ devices are a practical delivery method capable of producing a clinically relevant broader and deeper distribution of vaccines to the nasal respiratory mucosa, areas rich in dendritic cells and aggregates of lymphoid tissue, offering potential for a range of vaccines to produce improved immune response in non-parenteral delivery forms [24, 50].

## Assessment of nasal deposition and clearance—clinical aspects

### CFD simulations

With development of high-resolution CT and MRI technology, it has become possible to generate accurate 3D reconstructions of the complex nasal anatomy (Fig. 3). The field of computational fluid dynamics (CFD) is rapidly progressing in medicine and has enabled CFD simulations of nasal aerodynamics and deposition patterns [98–101]. The greatly improved density of the grids used and algorithms, along with much faster computers available for simulation, now allow implementation of more realistic conditions. For example, recent publications describe algorithms to simulate septal abnormalities, post-surgical changes, as well as heat and water exchange, and to more accurately simulate the true properties of aerosol generation and plume characteristics [99–101]. Undoubtedly, as the quality and capabilities increase, CFD simulations will play an increasingly important role and allow for realistic simulation of nasal physiology and drug delivery. A more detailed review of this exciting field is outside the scope of this review.

### Deposition studies in casts

The progress in imaging and reconstruction software has also made it possible to make physical models in rigid materials by modern 3D printing techniques like stereolithography with correct nasal geometry and dimensions. Casts made in softer material like silicone may offer advantages in terms of more realistic device cast interface. However, caution is necessary because even the softer silicone casts do not realistically represent the nasal valve dynamics, the cyclic physiological changes of the mucosa, or reflect the in vivo surface properties of the nasal mucosa, including the impact on mucocliliary clearance [102].

An in depth review of in vitro drug delivery simulation performed in nasal casts is also outside the scope of this review, but some comments related to recent work are included to highlight issues related to the interpretation and predictive value of results obtained with nasal delivery devices in cast studies. Three recent publications report in detail on the effect of breathing patterns, formulation, spray pump variables, and the site of deposition in a particular commercially available silicone cast (Koken Co., Japan) [65, 103, 104]. An interesting gel coating method that changes color in contact with the liquid allowing quantification of deposition by photometric analysis of deposition images is described [103]. In the most recent work, different insertion depth, spray angle, and plume characteristics (cone angle and particle size distribution) were studied. Data on the dimensions of the cast are not presented in these reports; however, it is critical to note that the Koken cast is, according to the manufacturer, primarily an educational tool and that it therefore has a flat transparent septum to enable visualization of complicated nasal structures. Inspection of the nasal valve area and objective measurements of the dimensions reveals that the dimensions at the valve area are several-fold larger than the average human valve dimensions and outside the normal range [105]. It is suggested in these recent publications that casts studies have potential for establishing in vivo bioequivalence and as indicators of critical quality attributes [65]. While an admirable goal, the lack of validation of all cast dimensions coupled with the inability of the cast to reproduce important dynamic aspects of nasal anatomy and physiology discussed previously, certainly casts doubt on the ability to achieve this objective with the Koken cast, and potentially any rigid nasal cast. Nevertheless, the use of ever-improving casts coupled with innovative techniques such as photometrics may be very useful in development of new nasal delivery devices. Reliance on standards published by FDA for performance of spray pumps may seem appropriate for comparison of nasal delivery devices; however, published analysis also suggests that the in vitro measurements in the FDA guidance related to performance of spray pumps are not clinically relevant [2]. Thus, in light of current methodological and technological limitations, human in vivo deposition and clearance studies, and relevant human clinical trials, allowing regional deposition quantification and direct clinical comparisons, respectively, are still ultimately required. A recent review concludes that although both in vitro studies and in vivo imaging methods may be of value during the device development stages, ultimately, randomized placebo-controlled trials quantifying both symptoms and functional parameters are required to determine drug delivery efficiency of different devices [42].

### In vivo assessment of deposition and clearance

A number of gamma deposition studies, a study using radiopaque contrast, and studies using colored dyes confirm that administration with conventional spray pumps, pMDIs, nebulizers, and powder devices all result in deposition mainly in the anterior non-ciliated segments of the nose anterior to and at the narrow nasal valve, which is regarded suboptimal for clinical efficacy where deep and broad nasal deposition is required [13, 43, 63, 66, 72, 79, 106]. Colored dyes may offer a quick and inexpensive semi-quantitative assessment of deposition and clearance, and a number of studies have assessed deposition patterns with dyes with the goal of improving deposition and the clinical outcome of delivery with spray pumps and drops [43, 107, 108]. Although results vary, the effect of different body positions and administration techniques appears to have limited impact on initial deposition patterns. In fact, a recent single-blind, cross-over study comparing seven different administration techniques of colored dyes in healthy individuals using endoscopic video imaging concluded that there may not be a single “best” technique for topical nasal drug delivery with conventional nasal sprays [108]. Lack of patient compliance further reduces the clinical usefulness of these delivery techniques.

More detailed assessment of drug deposition using regional gamma-deposition patterns have added to the understanding of deposition and clearance patterns and how they may have an impact on the clinical outcomes [13, 14, 66, 70, 72]. Improved methods for positioning and re-positioning of the test subjects and the use of radiolabeled gases and MRI overlay allow regional quantification of nasal deposition and outcomes [66, 70]. Furthermore, in contrast to earlier studies, proper correction for regional differences in tissue attenuation in the different nasal segments and between the nose and lungs is now being performed [13, 14, 70]. This review only addresses in vivo gamma-deposition studies dealing with some key aspects related to the in vivo performance of nasal delivery devices that normally get limited attention.

### Impact of delivery instructions, patient compliance, and body position

One factor too often neglected when comparing deposition studies is whether the delivery procedure was performed by the subjects themselves or by an assistant. Clearly, delivery by the subjects is much closer to the real-life situation, but inevitably introduces more variability. In most gamma-deposition studies, a trained assistant inserts the spray device and performs the actuation according to a strict protocol. This was the case in a study assessing deposition of radiolabeled cromoglycate substantial delivery beyond the nasal valve along the nasal floor was observed [109]. In contrast, in a study with radiolabeled insulin where the spray was actuated by the subjects themselves, it was noted that individual administration technique resulted in the majority of doses being deposited in the anterior rather than the posterior nasal cavity in five out of six subjects, with the dose then being cleared via the nares rather than the nasopharynx [110]. Contrary to expectations, no sign of systemic absorption of insulin was observed, and the authors commented that this effect of individual administration technique raises a separate question on the usefulness of nasal spray doses for delivery of insulin intended for systemic absorption [110].

### Overall versus regional clearance patterns

Gamma studies must be performed in a controlled setting where subjects are more likely to adhere to instructions for use of the devices than in real life. It is very common to observe that subjects during, or immediately after, administration of drug using nasal devices intuitively sniff to avoid the concentrated anterior liquid deposition from dripping out and down on the upper lips. Sometimes, the anteriorly deposited surplus is wiped off, as has been observed in gamma-deposition studies [111]. In fact, considerable early drip-out has been observed in a gamma study following self-administration with a 100-μl standard nasal spray pump, which causes concentrated anterior deposition. This phenomenon has also been observed after delivery with nebulizers [14, 72]. Recent studies offering regional clearance curves for four or six nasal segments highlight that the initial site of deposition has a major impact on the clearance rates and that determination of overall nasal clearance is a very crude and potentially misleading measure that does not predict clinical performance [13, 14]. Interestingly, a recent review on pulmonary drug delivery states that total lung deposition appears to be a poor predictor of clinical outcome; rather, regional deposition needs to be assessed to predict therapeutic effectiveness [112]. In a study comparing nasal deposition and clearance after self-administration of the same conventional spray pump (100 μl) by hand in the traditional way and by breath actuation with a Bi-Directional™ delivery device (see below for details), the percentage left in the nose 30 min after hand actuation is twice that of breath actuation (46 % vs. 23 %). However, the regional deposition patterns (divided in four nasal segments) reveal that this difference is primarily a result of anterior retention in the predominantly non-ciliated anterior two nasal quadrants following hand-actuated spray delivery. The deposition pattern is reversed with the Bi-Directional™ device, which was reported to offer three times greater broader and more reproducible deposition to the ciliated respiratory mucosa beyond the nasal valve and, in particular, in the upper posterior segments, with removal at a speed corresponding to expected mucocliliary clearance rate [13]. Another study comparing self-administration of a spray pump and a Bi-Directional™ breath-actuated powder device showed a similar significant difference in the regional deposition and clearance patterns, further reinforcing the importance of evaluating not only overall or “whole-nose” deposition and clearance but instead also evaluating regional patterns when developing or comparing nasal delivery devices [14] (Fig. 4).

### Impact of site of delivery and volume on deposition and clearance

The results from the study described above comparing deposition and clearance after delivery from the same spray pump actuated in different manners show that the initial site of deposition has a profound impact on the clearance rates [3, 13, 14]. Interestingly, McLean et al. [113] described three different phases of nasal clearance.

1. The first phase occurs within the first minute after administration and is particularly evident following delivery of large concentrated volumes that rapidly pass along the floor of the nose to the pharynx to be swallowed. This applies in particular to delivery of drops and can contribute to explaining the much lower absorption of desmopressin delivered as drops, but also applies to spray delivery with higher spray volumes [3, 14, 51, 113]. The initial and very rapid removal may not always be recognized, as the initial gamma image often includes averaging of registration of counts over a 2-min period due to the relatively small dose of radioactivity used (for ethical reasons) [14].
2. The second phase lasts for about 15 min and corresponds to mucociliary clearance of the fraction initially deposited on the ciliated respiratory mucosa found at and beyond the nasal valve [3, 13, 14, 51, 63, 70, 113, 114].
3. The third prolonged late phase represents the slow removal of residual drug deposited in the anterior non-ciliated parts of the nasal surface and can take hours, unless mechanically removed by nose blowing and/or wiping of the nose [63]. Consequently, depending on whether the substance in question has local action, is intended for systemic absorption, for N2B transport, or a combination, the primary goal is frequently to maximize exposure to the ciliated mucosa beyond the nasal valve. One strategy for enhanced exposure is to slow clearance by thixotropic or bioadhesive agents or agents which slow ciliary action in order to increase the residence time in this region or by adding absorption enhancer if systemic absorption is the objective [78, 115].

In principle, an alternative, complementary, and probably better way to enhance the exposure is to modify/improve the administration method or technique. The goal should be to reduce the amount of drug quickly passing through the nose to be swallowed in the first phase, to reduce the amount deposited outside the nose, and to increase the amount bypassing the nasal valve and the nasal respiratory mucosal surface covered. Delivery of smaller particles with a traditional spray offers advantages in terms of absorption and biological response compared to delivery of drops, and repeated delivery of a smaller volume, as 2 × 50-μl spray has been reported to be better than 1 × 100 μl for systemic absorption [51, 114]. In contrast, another study found that spraying 1 × 100 μl resulted in larger deposition than 2 × 50 μl beyond the nasal valve with more rapid overall clearance, but the study did not assess absorption or biological response [63]. A narrow cone angle resulted in more posterior deposition and faster clearance than a cone of 60°, and drops deposited more posteriorly are cleared faster [116, 117] .

For locally acting anti-inflammatory drugs like steroids and antihistamines, as well as for vaccines, the non-ciliated surface of the vestibule is not the target [42]. However, recent publications continue to advocate concentrated anterior deposition and retention as desirable and a key advantage of the novel HFA-based nasal pMDI with topically acting drug [118]. Reference is made to a paper from 1987 with CFC-based pMDI showing that as much as 65 % of the initial radioactivity is retained in the anterior parts of the nose after 30 min and incorrectly stating that an almost total clearance was observed 30 min after delivery with aqueous spray [63]. A recent publication even claims that the anterior retention following pMDI delivery provides evidence for enhanced efficacy, which seems to be in conflict with the prevailing opinion [42, 118].

## Conclusions

The nose is attractive for delivery of many drugs and vaccines, but the potential has not been fully realized. Inherent challenges related to the nasal anatomy, physiology, and aerodynamics that may severely limit the potential and clinical efficiency are not widely understood. The small and dynamic dimensions of the nasal cavity and the anterior anatomy are among the most important hurdles for more efficient nasal drug delivery. Despite important improvements in the technical device attributes that can offer more reproducible and reliable in vitro performance, this has to a limited extent translated into improved clinical performance. While in vitro performance testing is undoubtedly of value for product quality assessment, predictive value for in vivo clinical performance is highly questionable [2]. CFD simulations of nasal aerodynamics and cast studies may be of value in the developmental stages of device design, and future advances may improve their predictive value. Human in vivo deposition and clearance studies can be very important, providing valuable information particularly if recent advances allowing regional quantification and tissue attenuation correction are employed [14, 70, 112]. Still, delivery by trained assistants in controlled environments may not adequately reflect the device performance in the clinical setting. Even the most advanced nebulizer technologies introduced have shown poor delivery efficiency, with undesirable localized delivery in the non-ciliated anterior nasal region and along the floor of the nose and problems with inhalation exposure of the lungs [72]. As stated in a recent review, well-controlled clinical studies are currently required to quantify changes in both symptoms and functional parameters, and ultimately to determine the efficacy of novel drug/device combinations [42]. The Bi-Directional™ drug delivery concept introduces a novel approach that can overcome inherent limitations of conventional nasal delivery imposed by the dynamics of the nasal valve. Gamma-scintigraphy studies with both powder and liquid Bi-Directional™ device variants confirm significant improvements in regional in vivo deposition and clearance patterns, and a number of clinical trials suggest that this deep nasal deposition translates into clinical benefits for the patients. This delivery technology can be combined with a variety of dispersion technologies for both liquids and powders, and promises to expand the possibilities of nasal drug delivery.
